# Co-Targeting of BTK and TrxR as a Therapeutic Approach to the Treatment of Lymphoma

**DOI:** 10.3390/antiox12020529

**Published:** 2023-02-20

**Authors:** Sicong Wang, Erin Clapper, Kathryn F. Tonissen, Giovanna Di Trapani

**Affiliations:** 1 School of Environment and Science, Griffith University, Nathan, QLD 4111, Australia; 2 Griffith Institute for Drug Discovery, Griffith University, Nathan, QLD 4111, Australia

**Keywords:** thioredoxin reductase, bruton’s tyrosine kinase, B-cell receptor signaling pathway, DLBCL

## Abstract

Diffuse large B-cell lymphoma (DLBCL) is a haematological malignancy representing the most diagnosed non-Hodgkin’s lymphoma (NHL) subtype. Despite the approved chemotherapies available in clinics, some patients still suffer from side effects and relapsed disease. Recently, studies have reported the role of the Trx system and the BCR signalling pathway in cancer development and drug resistance. In this regard, we assessed a potential link between the two systems and evaluated the effects of [Au(d2pype)_2_]Cl (TrxR inhibitor) and ibrutinib (BTK inhibitor) alone and in combination on the cell growth of two DLBCL lymphoma cell lines, SUDHL2 and SUDHL4. In this study, we show higher expression levels of the Trx system and BCR signalling pathway in the DLBCL patient samples compared to the healthy samples. The knockdown of TrxR using siRNA reduced BTK mRNA and protein expression. A combination treatment with [Au(d2pype)_2_]Cl and ibrutinib had a synergistic effect on the inhibition of lymphoma cell proliferation, the activation of apoptosis, and, depending on lymphoma cell subtype, ferroptosis. Decreased BTK expression and the cytoplasmic accumulation of p65 were observed after the combination treatment in the DLBCL cells, indicating the inhibition of the NF-κB pathway. Thus, the co-targeting of BTK and TrxR may be an effective therapeutic strategy to consider for DLBCL treatment.

## 1. Introduction

Lymphoma refers to a group of cancers that arise from lymphocytes and is considered to be the most common form of haematological malignancy in adults. Diffuse large B-cell lymphoma (DLBCL) originates from B-cells and is considered the most common type of non-Hodgkin’s lymphoma (NHL) [1,2,3,4,5]. DLBCL can be further categorised into two subtypes according to their molecular features: the germinal centre B-cell (GCB) and the activated B-cell (ABC) DLBCL subtypes [6]. Although R-CHOP (cyclophosphamide, doxorubicin, vincristine, prednisone, and rituximab) is the standard chemotherapy regime used to treat DLBCL [7], approximately 40% of patients suffer from relapsed or drug-resistant DLBCL [8], with cardiotoxicity being the main side effect observed during treatment [9].

The B-cell receptor (BCR) signalling pathway is essential for B-cell survival and development. Under healthy physiological conditions, the activity levels of BCR signalling depends on the different stages of B cells [10,11]. However, the chronic activation of the BCR signalling pathway has been reported in different types of lymphomas, including DLBCL [12,13,14]. The BCR signalling pathway activates a range of downstream pathways, including NF-κB, ERK/MAPK, and AKT, which are all involved in cell proliferation and differentiation [15,16,17]. These pathways lead to increased cell growth and help cells escape from death. Bruton’s tyrosine kinase (BTK) is the key protein that links BCR activity to those downstream pathways [18]. Ibrutinib is an FDA-approved irreversible covalent BTK inhibitor that has been used to clinically treat chronic lymphocytic leukemia (CLL) and mantle cell lymphoma (MCL) [19]. Several clinical trials are undergoing with regard to different types of lymphoma, including DLBCL (NCT01325701), relapsed MCL (NCT02169180), follicular lymphoma (NCT02451111), and double-hit B-cell lymphoma (NCT02272686). However, ibrutinib can also target other kinases, including interleukin-2-inducible T-cell kinase [20] and tec protein tyrosine kinase [21]. Furthermore, patients treated with ibrutinib suffer from cardiac side effects, such as an increased risk of atrial fibrillation, and bleeding resulting from platelet disfunction [22].

The thioredoxin (Trx) system comprises thioredoxin (Trx), thioredoxin reductase (TrxR), and NADPH [23]. This system is one of the main antioxidant systems that regulate redox homeostasis by maintaining the levels of reactive oxygen species (ROS) in cells [24]. It also participates in several signalling pathways, including the NF-κB pathway [25,26], by regulating the disulphide bonds of target proteins. The upregulation of the Trx system and the NF-κB pathway in lymphoma patients has been reported, which is associated with cancer cell survival [27]. Recent studies suggest that targeting the Trx system could result in cancer cell apoptosis via inhibiting several pathways [28,29,30]. Studies have shown that auranofin, an FDA-approved drug for treating rheumatoid arthritis, could also inhibit cancer cell development via inhibiting the activity of TrxR. However, auranofin was found to target other proteins, including proteasomal proteins, 3-hydroxy-3-methyglutaryl-coenzyme A reductase (HMGCR), and cysteine and histidine-rich domain-containing protein 1 (CHORDC1) [31,32]. Another gold-based compound, [Au(d2pype)_2_]Cl, which is a more specific TrxR inhibitor than auranofin [33,34], has been shown to inhibit the proliferation of blood cancer cells in vitro [29,35,36]. In addition, previous studies in our lab have shown that [Au(d2pype)_2_]Cl inhibited lymphoma cell proliferation at a lower concentration than auranofin [35].

Since both the BCR signalling pathway and the Trx system participate in lymphoma survival and play a crucial role in lymphoma development, our aim was to investigate the potential interaction of the Trx system and the BCR signalling pathway and to provide a better understanding of the role of the two systems in lymphoma. Targeting these two systems together by using low concentrations of their specific inhibitors may result in cancer cell death and fewer side effects. Hence, investigating the relationship between the Trx system and the BCR signalling pathway may provide insight into potential new therapeutic strategies for lymphoma treatment.

## 2. Method and Materials

### 2.1. Cells and Reagents

SUDHL2 (ATCC^®^ CRL-2956™) and SUDHL4 (ATCC^®^ CRL-2957™) cell lines were procured as a kind gift from Professor Maher Gandhi (Princess Alexandra Hospital, Brisbane, Australia). They were authenticated by the Griffith University DNA Sequencing Facility (GUDSF), (Griffith University, Brisbane, QLD, Australia) using the STR profiling method (GenePrint^®^ 10 System, Promega, Madison, WI, USA). Both lymphoma cell lines were cultured in RPMI-1640 medium (Gibco, Gaithersburg, MD, USA) containing 10% (*v*/*v*) foetal bovine serum (FBS) (Bovagen, France), 200 mM of L-glutamine, 100 U/mL of penicillin, and 100 µg/mL streptomycin (Gibco, Gaithersburg, MD, USA). RT-qPCR oligonucleotides were purchased from Integrated DNA Technologies (IDT, Singapore). The anti-β-actin (sc-47778) and anti-BTK (sc-28387) antibodies were obtained from Santa Cruz Biotechnology (Santa Cruz, CA, USA); the anti-TrxR1 (cat no. MAB7428) was purchased from R&D Systems (Minneapolis, MN, USA); and the anti-vinculin (cat no. 13901) was obtained from cell signalling Technology. [Au(d2pype)_2_]Cl was gifted by Professor Sue Berners-Price (Glycomics, Griffith University, Southport, QLD, Australia) and obtained as described previously [37]. Ibrutinib, Z-VAD(OMe)-FMK, and ferrostatin-1 were purchased from Cayman Chemicals (Ann Arbor, MI, USA).

### 2.2. Compounds Preparation

Ibrutinib, Z-VAD(OMe)-FMK, and ferrostatin-1 were prepared in 100% DMSO at a concentration of 64 mM and were diluted in 1× PBS. [Au(d2pype)_2_]Cl was prepared in 0.1% ethanol at a concentration of 300 μM and was diluted in phenol red-free RPMI-1640 medium.

### 2.3. Cell Proliferation Assay

Cells at a density of 40,000 cells/100 μL were seeded into a 96-well plate in triplicate. Media was used as blank. Cells were treated with increasing concentrations of drugs and incubated for 24, 48, or 72 h at 37 °C. Then, 10 µL of filtered sterile 5 mg/mL 3-(4,5-Dimethylthiazol-2-yl)-2,5-Diphenyltetrazolium Bromide (MTT) was added to each well and incubated for 3 h at 37 °C. Subsequently, 25 µL of 0.01 M HCl/20% (*w*/*v*) SDS was added to each well and incubated overnight at 37 °C. The plate was read at 570 nm in the FLUOstar Omega plate reader (BMG Lab-tech, Ortenberg, Germany) the next day.

### 2.4. Isobologram Analysis

To identify whether ibrutinib and [Au(d2pype)_2_]Cl cotreatment would exert an additive or synergistic effect on cancer cell cytotoxicity, an isobologram analysis was performed using MTT proliferation data. Two sets of concentrations were used for each compound. Firstly, a range of concentrations was based on the IC_50_ of each compound, and comprised 1-, 0.75-, 0.5-, 0.25-, 0.125-, and 0.0625-fold of the IC_50_. Secondly, a broader range of concentrations was selected for each compound according to the MTT cell proliferation assay results. Each concentration in the IC_50_ range of ibrutinib was combined with each concentration in the broader range of [Au(d2pype)_2_]Cl, and vice versa. The IC_50_ of each individual combination was then calculated and used in the isobologram analysis. The type of interaction occurring between ibrutinib and [Au(d2pype)_2_]Cl was identified by the fractional inhibitory concentration (FIC) method. The ΣFIC values were considered as synergistic (ΣFIC < 1), additive (ΣFIC = 1), or antagonistic (ΣFIC > 1) [38]. The interaction factor (I) was determined using SAAM II software (SAAM Institute, Seattle, WA, USA), where I < 0, I = 0, and I > 0 indicated antagonistic, additive, and synergistic, respectively.

### 2.5. TrxR Activity Assay

TrxR activity assays were performed as described previously [29]. Briefly, TrxR activity was measured in a SpectraMax M3 plate reader (Molecular Devices, VIC, Australia) based on the NADPH-dependent reduction of DTNB. Cells were lysed in 0.5% (*v*/*v*) Nonidet P-40 lysis buffer (150 mM of NaCl, 50 mM of Tris-Cl, 0.5% (*v*/*v*) Nonidet P-40, 0.5 mM of EDTA at pH 8, 2 mM of PMSF, and 1 μL/mL protease inhibitor cocktail VI (Astral Scientific, Sydney, NSW, Australia)). To excluded non-TrxR-specific DTNB reduction, cell lysates were treated with or without 8 μM of auranofin at room temperature for 30 min. The TrxR activity was determined using a buffer with 125 mM of potassium phosphate at pH 7.5, 2.5 mM of EDTA, 0.25 mM of NADPH, and 3.125 mM of DTNB. TNB production was monitored for 10 min at 412 nm, and rates were calculated from the linear portion of the graph. The specific TrxR activity (mU/mg protein) was computed by normalizing the units of TrxR activity with the protein levels in each sample (as determined using the BioRad DC protein assay (BioRad, NSW, Australia) as per manufacturer’s instructions).

### 2.6. Caspase-3 Activity Assay

Caspase activity was measured in untreated and treated lymphoma cells using Ac-DEVD-AMC (Cayman Chemical Company, Ann Arbor, MI, USA), as previously described [39]. Briefly, cells were seeded into a 24-well plate and treated with [Au(d2pype)_2_]Cl, ibrutinib, or a combination of [Au(d2pype)_2_]Cl/ibrutinib for 24 h. Cells were resuspended in 20 µL of 1× PBS after being washed twice with 1× PBS. Cell suspension was added into a black, clear-bottomed 96-well plate alongside 80 µL of caspase-3 buffer (5 mM of DTT, 100 mM of HEPES, pH 7.5, 10% (*w*/*v*) Sucrose, 0.1% (*v*/*v*) Nonidet P-40, and 50 µM of Ac-DEVD-AMC (Cayman Chemical Company, Ann Arbor, MI, USA)). The plate was immediately incubated in the FLUOstar Omega plate reader (BMG Labtech, Ortenberg, Germany) for 15 min at 37 °C, and AMC was determined with a fluorescence excitation wavelength of 370 nm and emission wavelength of 445 nm.

### 2.7. Transient Transfections

SUDHL2 and SUDHL4 cells (3 × 10^6^) were collected and washed with pre-transfection RPMI-1640 medium (without FBS or antibiotics). Cells were resuspended in 100 µL of pre-transfection RPMI-1640 medium and transferred into Eppendorf tubes. Then, 100 nM of TrxR1 siRNA molecules or control siRNA (IDT, Singapore) was added into separate tubes and incubated for 5 min at room temperature. Cells were transferred into transfection cuvettes and transfected using the Amaxa Nucleofector (P-005 program) (Lonza, Basel, Switzerland). After cells were incubated for a further 5 min at room temperature, cells were transferred into a 24-well plate in a post-transfection RPMI-1640 medium (with 10% FBS and no antibiotics) and incubated for 48 h at 37 °C before further experiments were conducted.

### 2.8. Reverse Transcriptase-Quantitative PCR (RT-qPCR)

Total RNA was extracted from SUDHL2 and SUDHL4 lymphoma cells using TRIsure™. Total RNA Lysis solution (Bioline, Sydney, NSW, Australia) according to the manufacturer’s instructions. The cDNA was synthesized from total RNA using the GoScript™ Reverse Transcription Mix (Promega, Madison, WI, USA). RT-qPCR was performed using the SensiFAST™ SYBR^®^ No-Rox Kit (Bioline, Sydney, NSW, Australia). The oligonucleotides (Integrated DNA Technologies, Singapore) used for RT-qPCR are listed in Table 1.

The following reaction conditions were used: 95 °C for 2 min followed by 40 cycles of 95 °C for 10 s, 60 °C for 15 s, and 72 °C for 20 s. Quantification was performed using Bio-Rad CFX96 Real-Time PCR Detection System (Bio-Rad, Hercules, CA, USA). The relative mRNA expression was measured by using the comparative cycle threshold algorithm (∆∆Ct) method. The mRNA expression levels were normalized against the expression levels of ribosomal protein L32 (RPL32) [40].

### 2.9. Western Blot Analysis

Samples were loaded on Any KD™ Mini-PROTEAN TGX™ precast protein gels (Bio-Rad, Hercules, CA, USA) and electrophoresed. Then, proteins were transferred onto polyvinylidene difluoride (PVDF) membrane using the Tran-Blot Turbo system (Bio-Rad, Hercules, CA, USA) and detected using appropriate primary and secondary antibodies. The signal detection was enhanced using the SuperSignal™ West Pico PLUS Chemiluminescent Substrate (Thermo Scientific™, Waltham, MA, USA). Densitometry analysis was optimised using the BioRad Image Lab software package and normalized with the loading control.

### 2.10. Bioinformatics

The RNAseq data were obtained from the TCGA (https://portal.gdc.cancer.gov/, accessed on 1 January 2022) diffuse large B cell lymphoma database (Project ID: TCGA-DLBC). Data were unified by the Toil process and analysed using UCSC XENA (https://xenabrowser.net/datapages/, accessed on 1 January 2022). Correlations between the Trx system genes and the BTK signalling pathway genes were analysed using Spearman’s rank-order correlation. The Spearman’s rank correlation coefficient (r) value ranged between −1~0 and 0~1, which were considered to correspond to either a negative or a positive correlation, respectively. R studio (version 3.6.3) (https://www.r-project.org/, accessed on 1 January 2022) was used for statistical analysis and visualization. The raw Fragments Per Kilobase Per Million (FPKM) data format was converted to Transcripts Per Million reads (TPM) format. Then, the TPM format data were transformed and reported as log_2_ values.

### 2.11. Human Protein Atlas

The Human Protein Atlas (https://www.proteinatlas.org, accessed on 1 January 2022) contains immunohistochemistry-based expression data for different cancers [41]. Direct comparison of protein expression of thioredoxin reductase and BTK between human normal and lymphoma tissues was carried out by assessing immunohistochemistry images.

### 2.12. Statistical Analysis

All values (*n* = 3, unless otherwise indicated) are displayed as mean ± SEM. Data in this paper were analysed by Graphpad Prism 9 software (GraphPad, San Diego, CA, USA) or R studio (version 3.6.3). Data with two groups and one parameter were analysed using a *t*-test followed by Students’ t distribution. A one-way ANOVA followed by Dunnet’s multiple comparisons test was used for data with three or more groups and one parameter. Data with more than two groups and more than one parameter were analysed by two-way ANOVA followed by Sidak’s test. *p* < 0.05 was considered significant.

## 3. Results

### 3.1. The Trx System and the BCR Signalling Pathway-Related Genes Are Overexpressed in DLBLC

**Figure 1:**
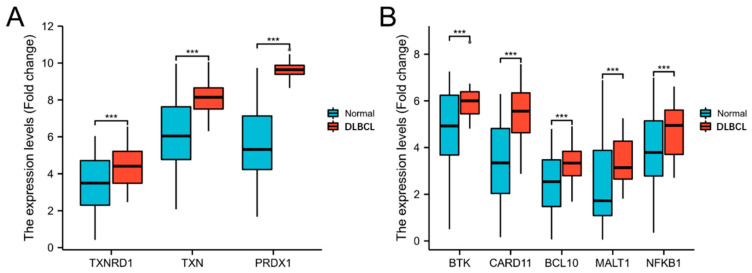
Trx system and BCR signalling pathway-related gene expression in DLBCL and healthy samples. (**A**,**B**), mRNA expression in diffuse large B-cell lymphoma (DLBCL) patients (*n* = 47) and healthy samples (*n* = 444) was analysed. The significance levels are marked as *** *p* < 0.001.

To determine whether the cytosolic Trx system and BCR signalling pathway genes are overexpressed in lymphoma patients’ cells, publicly available datasets were used. An analysis of the RNA-seq dataset from TCGA showed that the expression levels of genes related to the Trx system and the BCR signalling pathway were significantly upregulated in the DLBCL patient cells compared to the healthy samples (Figure 1A,B). In the DLBCL samples, the expression of TXNRD1 and TXN was approximately 1.3-fold and 2-fold higher than in the healthy samples, respectively. Peroxiredoxin 1 is a thiol-specific peroxidase that can regulate ROS in cells, and its activity is also dependent on the Trx system [42]. The expression of PRDX1 showed a four-fold upregulation in DLBCL compared to the healthy samples. The levels of all of the BCR signalling-related genes (BTK, CARD11, BCL10, MALT1, and NFKB1 (p50)) were significantly increased in the DLBCL samples by approximately 1.2- or 2-fold.

Since TrxR is a key protein that maintains the normal function of the Trx system and BTK is an essential protein that regulates the BCR signalling pathway, a qualitative analysis of the expression levels of these two proteins in the NHL samples and normal tissues was conducted using the Human Protein Atlas (Table 2 and Figure S1) (www.proteinatlas.org, accessed on 1 January 2022). NHL samples were analysed since they include DLBCL samples as well as other subtypes, with DLBCL being the main subtype. The results showed that TrxR protein expression was low in the normal lymph node tissue, whereas high expression levels of TrxR protein were detected in the NHL tissue samples. Furthermore, medium expression levels of the BTK protein were observed in the normal lymph nodes, while high expression levels were observed in the NHL samples. Overall, a higher expression of both proteins, TrxR and the BTK, was detected in NHL compared with normal lymph nodes.

### 3.2. The Gene Expression of TrxR Is Correlated with BCR Signalling Pathway-Related Gene Expression

**Figure 2:**
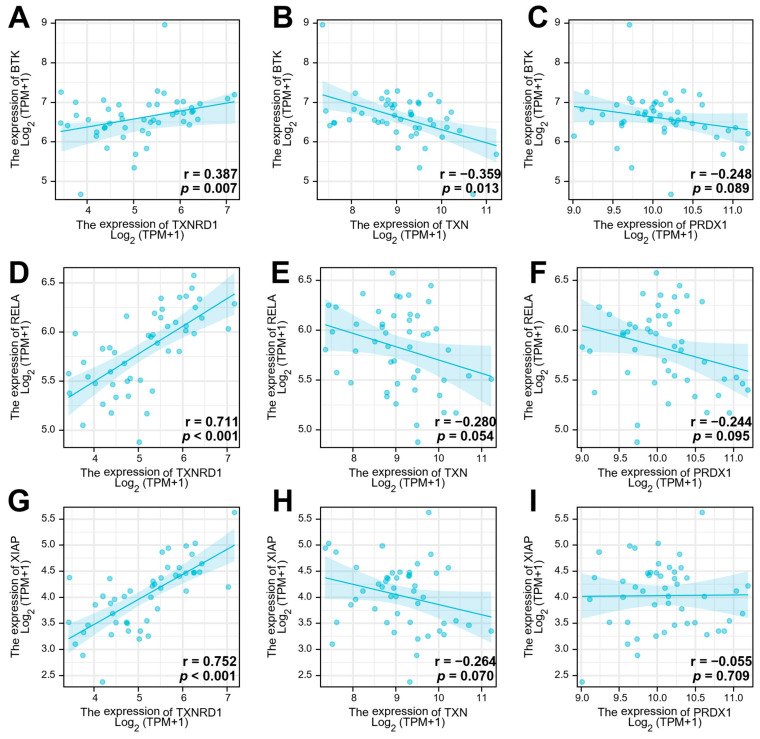
Correlation analysis of expression of genes of the Trx system and BCR signalling pathway. Expression of TXNRD1, TXN, and PRDX1 in comparison with BTK, RelA, and XIAP expression in DLBCL (*n* = 48). (**A**–**C**), correlation analysis between BTK and TXNRD1 (**A**), TXN (**B**), and PRDX1 (**C**); (**D**–**F**), correlation analysis between RelA and TXNRD1 (**D**), TXN (**E**), and PRDX1 (**F**); (**G**–**I**), correlation analysis between XIAP and TXNRD1 (**G**), TXN (**H**), and PRDX1 (**I**). The correlation coefficient^®^ was calculated using Spearman’s r correlation analysis, and *p* values are displayed.

Correlation analysis using Spearman’s rank-order correlation test was performed using the TCGA dataset to evaluate a possible relation between the Trx system and the BCR signalling gene expression in DLBCL. The results showed that TXNRD1 and BTK (r = 0.387 and *p* = 0.007; Figure 2A) were significantly positively correlated. The TXNRD1 and RelA (p65) expression also showed a significant positive correlation with a Spearman correlation coefficient of r = 0.711 (*p* < 0.001, Figure 2D). However, the correlation analysis indicated a negative correlation between TXN and BTK (r = −0.359, *p* = 0.013, Figure 2B) and no correlation between TXN and RelA (r = −0.28, *p* = 0.054, Figure 2E). Analysis of the expression of PRDX1 and BTK indicated that there was no correlation between those genes (r = −0.248, *p* = 0.089, Figure 2C). Similarly, there was no correlation between PRDX1 and RelA (r = −0.244 and *p* = 0.095; Figure 2F). The expression of XIAP, a downstream target gene of NF-κB, showed a significant positive correlation with TXNRD1 expression (r = 0.752, *p* < 0.001, Figure 2G) but no correlation with TXN (r = −0.264, *p* = 0.07, Figure 2H) or PRDX1 expression (r = −0.055, *p* = 0.709, Figure 2I). Overall, these results indicate that only TrxR1 expression was significantly correlated with the BCR signalling-related genes analysed in this study, suggesting a cross-talk between this protein and the BCR signalling pathway.

### 3.3. Knockdown of TrxR1 in Lymphoma Cells Decreased the Expression of BTK and Affected Its Downstream Signalling Pathway

**Figure 3:**
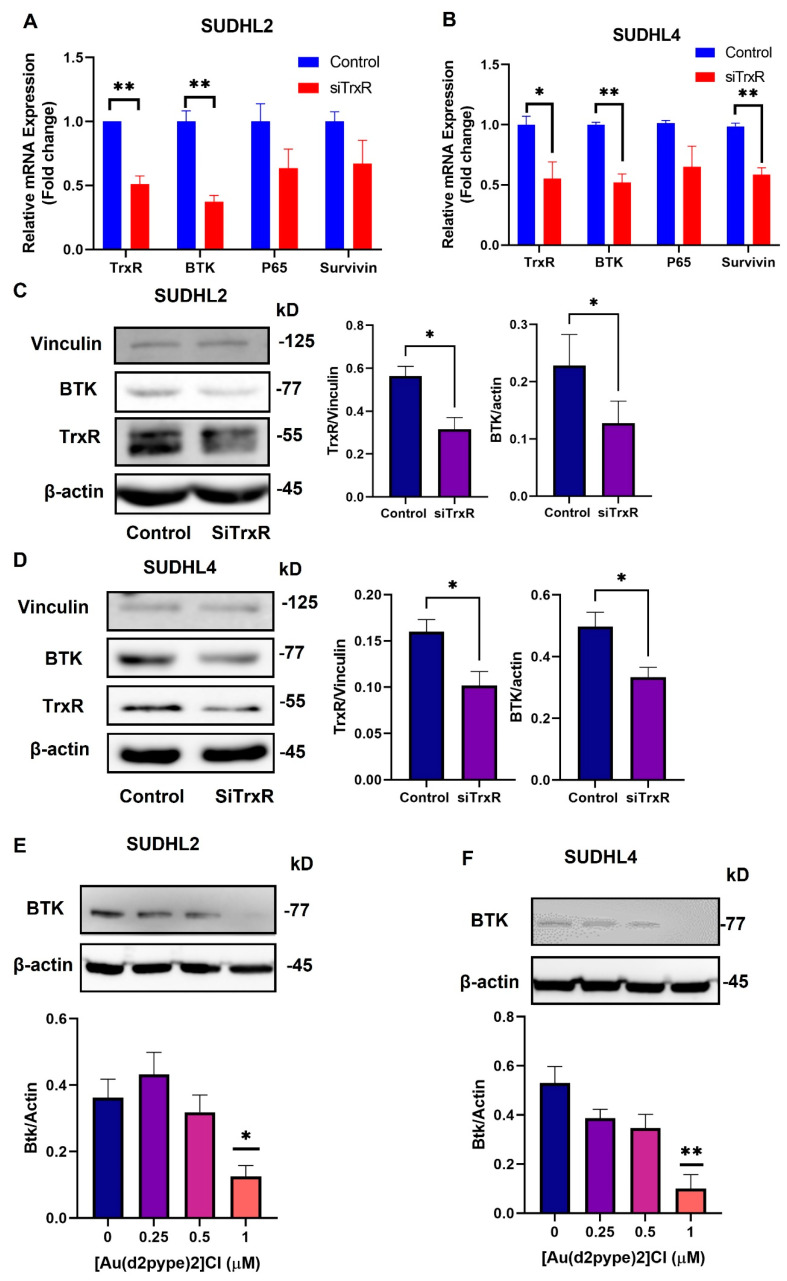
TrxR1 knockdown decreases BCR/NF-κB pathway-related mRNA and protein expression. SUDHL2 and SUDHL4 cells were transfected with TrxR1 specific siRNA (siTrxR) and a scrambled siRNA (control). (**A**,**B**), RT-qPCR was performed 48 h after transfection to determine the mRNA expression of TrxR1, BTK, p65, and surviving in SUDHL2 and SUDHL4 cells. (**C**,**D**), Western blotting was performed 48 h after transfection to determine the protein levels of BTK and TrxR in SUDHL2 and SUDHL4 cells. (**E**,**F**), The BTK expression was detected after treating cells with the indicated [Au(d2pype)_2_]Cl after 24 h in SUDHL2 and SUDHL4 cells. B-actin was used as the loading control for BTK protein blots, and Vinculin was used as the loading control for the TrxR protein blots. Western blots are representative of 3 independent experiments. Significance levels are marked as * *p* < 0.05 and ** *p* < 0.005.

To confirm that there was a positive correlation between TrxR and BTK in the SUDHL2 and SUDHL4 DLBCL cells, TrxR1 was knocked down using specific siRNA molecules. RT-qPCR and Western blotting confirmed that the knockdown of TrxR1 was successful (Figure 3A–D). The RT-qPCR results showed that, in the SUDHL2 cells, BTK mRNA expression levels were significantly decreased after the TrxR1 knockdown, while p65 and survivin mRNA expression was not significantly changed compared to the control (Figure 3A). In the SUDHL4 cells, the mRNA expression of BTK and survivin showed a significant decrease after the TrxR1 knockdown, while the p65 mRNA showed no significant difference (Figure 3B). These results indicated that by knocking down TrxR1 expression, there was a concomitant decrease in BTK expression, and supports the results observed in Figure 2, showing that the BTK mRNA expression was positively correlated with TrxR1 mRNA expression. In addition, the Western blotting results showed that BTK protein levels were also decreased after TrxR1’s knockdown (Figure 3C,D), suggesting that TrxR1 may regulate the expression of BTK in the SUDHL2 and SUDHL4 cells. In a previous study, [Au(d2pype)_2_]Cl, a TrxR inhibitor, was shown to inhibit TrxR and to reduce cell proliferation in lymphoma cells [35]. Therefore, [Au(d2pype)_2_]Cl was also used in this study to complement the siRNA experiment. Western blotting showed that a 1 mM [Au(d2pype)_2_]Cl treatment significantly reduced BTK protein levels in both the SUDHL2 and SUDHL4 cells (Figure 3E,F), indicating that the inhibition of TrxR activity by [Au(d2pype)_2_]Cl (Figure S2) could affect BTK expression, and may result in the downregulation of BCR-related signalling pathways, including the NF-κB pathway.

### 3.4. [Au(d2pype)_2_]Cl/Ibrutinib Cotreatment Synergistically Induced Cell Death in Lymphoma Cells

Previously, we showed that micromolar concentrations of [Au(d2pype)_2_]Cl significantly inhibited TrxR activity and cell growth in lymphoma cells [35], and this did not cause significant cell death in healthy peripheral normal blood mononuclear cells (PBMC) [29]. Ibrutinib is a specific BTK inhibitor that has been approved to treat CLL and MCL [19]. Several studies have shown that ibrutinib can inhibit NHL cell proliferation [43,44,45]. To determine the effect of the [Au(d2pype)_2_]Cl/ibrutinib cotreatment on lymphoma cells, MTT assays were performed using the two lymphoma cell lines treated with ibrutinib alone or in combination with [Au(d2pype)_2_]Cl. In this study, 0.25 mM [Au(d2pype)_2_]Cl was used, a concentration that has been shown to inhibit approximately 50% of TrxR activity (Figure S2) and to have a minimal effect on cell proliferation [35].

**Figure 4:**
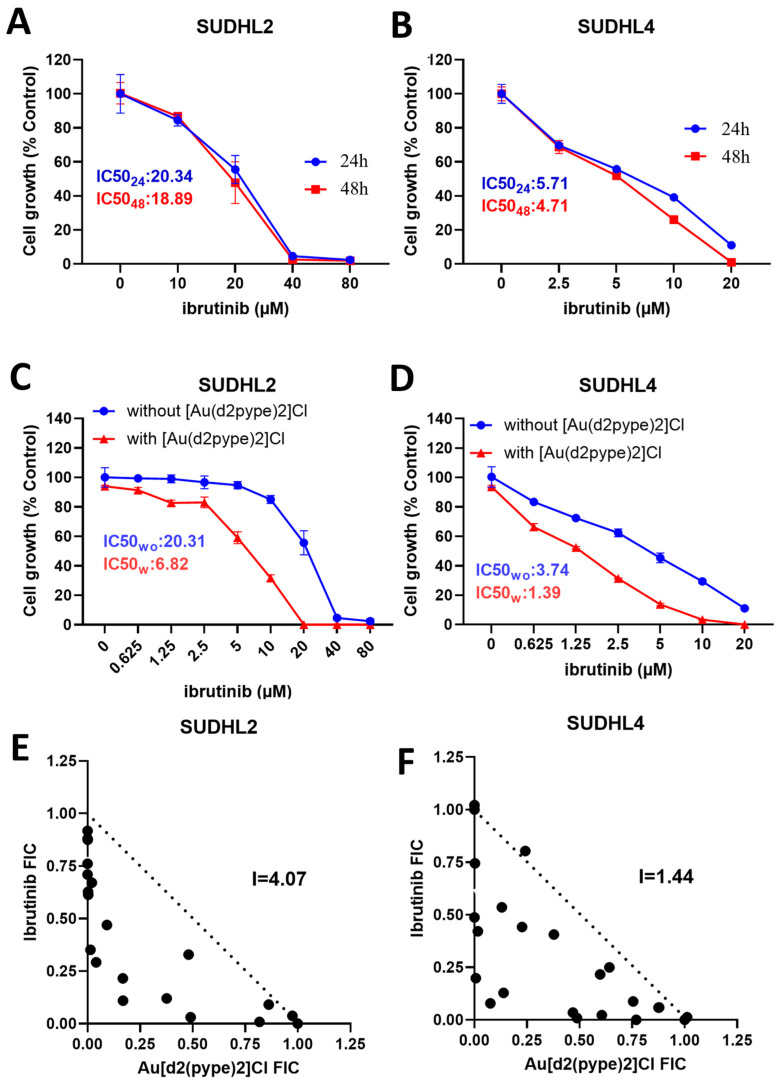
The cell proliferation and the isobologram analysis in DLBCL cell lines treated with ibrutinib with or without [Au(d2pype)_2_]Cl. SUDHL2 (**A**) and SUDHL4 (**B**) were treated with indicated concentrations of ibrutinib for 24 or 48 h. (**C**) SUDHL2 and (**D**) SUDHL4 cells were treated with ibrutinib alone or in combination with 0.25 µM of [Au(d2pype)_2_]Cl for 24 h. In the figures, the IC_50_ of ibrutinib alone is presented as IC_50wo_, and the IC_50_ of ibrutinib with [Au(d2pype)_2_]Cl is presented as IC_50w_. (**E**,**F**), isobolograms between [Au(d2pype)_2_]Cl and ibrutinib in the lymphoma cell lines. The plots showing the FIC values indicate the drug interaction and the dashed lines mark the FIC = 1. Two independent experiments with three technical replicates were performed in each lymphoma cell line.

We first examined ibrutinib’s dose and time responses in the SUDHL2 and SUDHL4 cell lines. The IC_50_ values of ibrutinib in the SUDHL2 cells were 20.34 µM (24 h) and 18.59 µM (48 h) (Figure 4A). However, the SUDHL4 cell line was more sensitive to ibrutinib, with IC_50_ values of 5.71 µM and 4.71 µM after 24 h or 48 h treatment, respectively (Figure 4B). Overall, the results showed that in regard to cell growth, the 24 and 48 h treatments had no significant differences in the lymphoma cells, which indicated that ibrutinib’s anticancer activity in these cell lines was mainly dose-dependent. In addition, based on the IC_50_ values, the SUHDL4 cells were more sensitive to ibrutinib than the SUDHL2 cell line.

To determine whether the [Au(d2pype)_2_]Cl and ibrutinib combination treatment had better anticancer activity than the ibrutinib treatment alone, combination assays (Figure 4C,D) and isobologram analysis (Figure 4E,F) were carried out for the lymphoma cell lines. The results showed that the IC_50_ of ibrutinib was significantly decreased when combined with [Au(d2pype)_2_]Cl in the lymphoma cell lines. The IC_50_ value of ibrutinib was 6.82 µM in the SUDHL2 cell line after the cotreatment, which can be compared with 20.31 μM when treated alone (Figure 4C). In the SUDHL4 cell line, the IC_50_ value of ibrutinib was 1.39 µM after the cotreatment, which can be compared with 3.74 μM when treated alone (Figure 4D). The IC_50_ value of ibrutinib used in the combination studies in the lymphoma cell lines was decreased by approximately two- to three-fold compared with the single ibrutinib treatment.

The 24 h [Au(d2pype)_2_]Cl/ibrutinib treatment data were used to plot the isobolograms and identify if the cotreatment had synergistic, additive, or antagonistic effects. The ΣFIC values of the two compounds in the two lymphoma cells were below 1, which indicated that [Au(d2pype)_2_]Cl and ibrutinib had a synergistic effect in the lymphoma cell lines (Figure 4E,F). In addition, the interaction factor (I) values were 4.07 and 1.44 in the SUDHL2 and SUDHL4 cells, respectively, thus confirming the synergistic effect of the two compounds (drugs) in the two cell lines. These results indicated that the combination of [Au(d2pype)_2_]Cl and ibrutinib had the most synergy in the SUHL2 cells compared with the SUDHL4 cells since the highest I value was calculated in the SUHL2 cells. These results also indicated that the [Au(d2pype)_2_]Cl/ibrutinib combination rendered the cells more sensitive than ibrutinib alone, and suggested that targeting the TrxR and BTK using [Au(d2pype)_2_]Cl and ibrutinib at the same time may constitute a new, efficient strategy against lymphoma.

### 3.5. Ibrutinib/[Au(d2pype)_2_]Cl Cotreatment Induced Cell Death via Apoptosis and Ferroptosis in Lymphoma Cells

**Figure 5:**
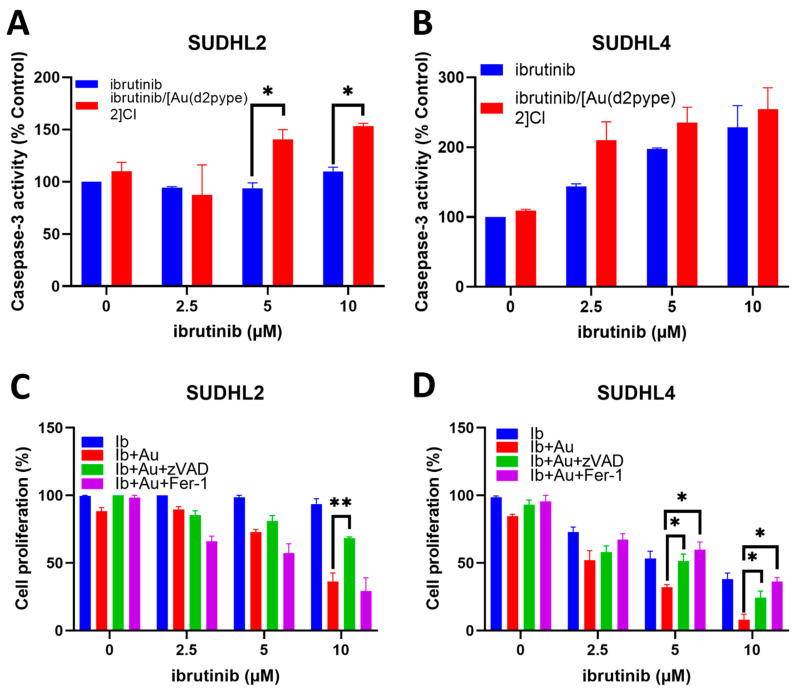
Caspase-3 activity and cell inhibition in DLBCL cell lines after treatment. (**A**,**B**), the two DLBCL cell lines were treated with the indicated concentrations of ibrutinib with or without [Au(d2pype)_2_]Cl for 24 h. (**A**) SUDHL2 and (**B**) SUDHL4 cells were cotreated with 0.25 µM of [Au(d2pype)_2_]Cl. (**C**,**D**), lymphoma cells were treated with the indicated concentrations of ibrutinib with or without [Au(d2pype)_2_]Cl plus 25 µM zVAD-fmk, or 10 µM Ferrostatin-1 for 24 h. Significance levels are marked as * *p* < 0.05 and ** *p* < 0.005.

Previous studies showed that [Au(d2pype)_2_]Cl induced blood cancer cell apoptosis via inhibiting TrxR activity and activating caspase-3 activity [29,35]. To investigate whether the caspase-3-dependent apoptosis pathway was induced by the ibrutinib/[Au(d2pype)_2_]Cl cotreatment, caspase-3 activity assays were performed in the lymphoma cell lines. The results showed that low concentrations (2.5–10 mM) of ibrutinib alone did not affect the caspase-3 activity in the SUDHL2 cell lines, while the ibrutinib/[Au(d2pype)_2_]Cl cotreatment boosted the activity of caspase-3 activity (Figure 5A). Significantly increased caspase-3 activity was observed after the SUDHL2 cells were treated with 5 µM or 10 µM of ibrutinib combined with 0.25 µM of [Au(d2pype)_2_]Cl compared with the ibrutinib alone treatment (Figure 5A). However, both the ibrutinib alone or the combination treatment in the SUDHL4 cells resulted in a visible increase in caspase-3 activity, although the cotreatment group showed higher, but not statistically significant, activity than the ibrutinib alone treatment at 2.5 µM and 5 µM, respectively (Figure 5B).

Apoptosis is one of the main cell death pathways that can be induced by chemotherapies in cancer cells. Ferroptosis is a newly discovered cell death pathway, and studies have reported that gold-based compounds can induce ferroptosis through the inhibition of antioxidant enzymes [46,47,48]. Therefore, the combination of ibrutinib and [Au(d2pype)_2_]Cl treatment might activate cell death pathways via either one or both of these mechanisms. To determine whether apoptosis and ferroptosis pathways were activated, zVAD, an apoptosis inhibitor, and ferrostatin-1, a ferroptosis inhibitor, were used. The results showed that in the SUDHL2 cell lines, the Fer-1 treatment was unable to rescue cells from cell death caused by the ibrutinib/[Au(d2pype)_2_]Cl treatment (Figure 5C). However, a significantly higher level of cell growth was observed in the SUDHL2 cells co-treated with 10 µM of ibrutinib and [Au(d2pype)_2_]Cl) in the presence of the apoptosis inhibitor zVAD compared to the cells only treated with ibrutinib combined with[Au(d2pype)_2_]Cl. In the SUDHL4 cells, a significantly higher level of cell growth was detected when the cells were treated with 5 µM and 10 µM ibrutinib combined with 0.25 µM of [Au(d2pype)_2_]Cl) in the presence of either of the two inhibitors compared to the cells only treated with ibrutinib combined with [Au(d2pype)_2_]Cl (Figure 5D). In contrast with the results observed in the SUDHL2 cell line, the Fer-1 cotreatment also significantly recovered the level of cell proliferation from the combination treatment of ibrutinib and [Au(d2pype)_2_]Cl in the SUDHL4 cells. These results indicated that the ibrutinib/[Au(d2pype)_2_]Cl treatment induced cell death mainly via the caspase-3-dependent apoptosis pathway in the SUDHL2 cells, while both apoptosis and ferroptosis pathways were activated in the SUDHL4 cells.

### 3.6. The [Au(d2pype)_2_]Cl/Ibrutinib Treatment Decreased the Total Expression of BTK in Non-Hodgkin’s Lymphoma Cell Lines

**Figure 6:**
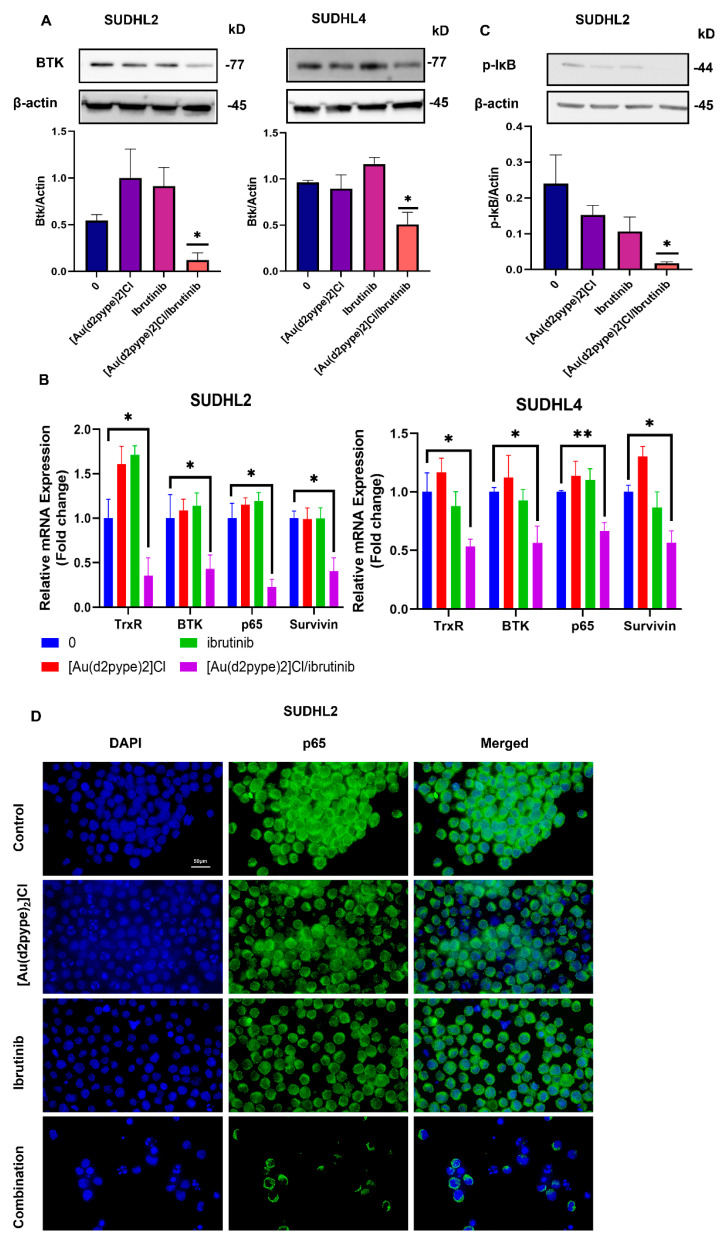
Expression of BTK and NF-κB signalling pathway after treatment. Cells were treated with 5 µM of ibrutinib (SUDHL4) or 10 µM of ibrutinib (SUDHL2) combined with 0.25 µM of [Au(d2pype)_2_]Cl. (**A**), protein levels of BTK were detected using Western blotting after 24 h treatment in SUDHL2 and SUDHL4 cells. (**B**), RT-qPCR was performed to detect the mRNA expression levels of TrxR, BTK, p65, and Survivin after 24 h in SUDHL2 and SUDHL4 cells. (**C**), Western blotting was carried out to determine the protein levels of p-IκB after 24 h treatment. (**D**), immunofluorescence staining was performed after 12 h with indicated treatment in SUDHL2 cells. Significance levels are marked as * *p* < 0.05 and ** *p* < 0.005.

The BCR signalling pathway plays an important role in the development of lymphoma, and BTK is a key protein that mediates signalling to the downstream pathways, including the NF-κB pathway [11]. Therefore, the expression levels of BTK were assessed in the SUDHL2 and SUDHL4 cells after their treatment with [Au(d2pype)_2_]Cl/ibrutinib to evaluate the effect of the combination treatment on the BCR signalling pathway. Western blotting analysis showed that treatment with [Au(d2pype)_2_]Cl or ibrutinib alone did not affect the expression of BTK. In contrast, the combination treatment significantly inhibited BTK protein expression (Figure 6A). The RT-qPCR results showed that there were no significant changes in TrxR1, BTK, p65, and survivin mRNA expression after ibrutinib or [Au(d2pype)_2_]Cl treatment in the SUDHL2 and SUDHL4 cell lines (Figure 6B). However, both TrxR1 and BTK mRNA expression were significantly reduced after the combination treatment in the SUDHL2 cells. The mRNA expression of p65, a subunit of NF-κB, and of survivin, whose mRNA expression levels can be regulated by NF-κB, also significantly decreased in the SUDHL2 cells after treatment (Figure 6B). In the SUDHL4 cells, a similar result was detected, and the TrxR1, BTK, p65, and survivin mRNA expression levels were significantly reduced after the combination treatment (Figure 6B).

Since decreased mRNA expression levels of p65 and survivin were obtained after the combination treatment (Figure 6B), Western blotting and immunofluorescence studies were performed to determine the effects of this treatment on the activation of the NF-κB pathway and the intracellular location of the p65 protein. The Western blotting results showed that the phosphorylation of IκB was significantly decreased in the cells after the combination treatment, while there was no significant difference when the cells were treated with [Au(d2pype)_2_]Cl or ibrutinib alone (Figure 6C). Since IκB is an inhibitor of NF-κB, and as it is released from NF-κB subunits after phosphorylation [49], the decreased phosphorylation of IκB indicated that NF-κB release was inhibited after the combination treatment. As a transcription factor, NF-κB can translocate to the nucleus and promote the expression of several survival factors [50], while the inhibited NF-κB would be retained in the cytoplasm. To determine whether the combination treatment induced NF-κB translocation, immunofluorescence studies were performed. The results in Figure 6D suggested that, without any treatment, the NF-κB was located in both the nucleus and cytoplasm. However, the NF-κB in the nucleus was decreased after either the [Au(d2pype)_2_]Cl or ibrutinib alone treatments, and the majority of NF-κB was located in the cytoplasm after the combination treatment. This result corresponded with the decreased levels of phosphorylation of IκB after cotreatment in the SUDHL2 cells. Taken together, these results indicated that the NF-κB signalling pathway was inhibited after the combination treatment.

## 4. Discussion

Despite the major achievements obtained with the current standard therapies for lymphoma treatment, long-term side effects, age-dependent responses to treatment, and relapsed/refractory disease due to resistant cancer cells continue to be major problems for lymphoma patients and, therefore, novel therapies need to be evaluated.

Redox balance, which participates in the regulation of several signalling pathways, including the BCR and NF-κB signalling pathways, is important for cancer cell development [51,52]. Cancer cells are heavily dependent on antioxidant molecules, including those of the thioredoxin system [53], to maintain this balance and counteract increased ROS production, which is usually detected in cancer cells due to their abnormal metabolic activity [54]. Hence, the targeting of antioxidant systems to disrupt the redox balance and push oxidative stress to a maximum cytotoxicity threshold has become a novel approach in cancer treatment [55].

Ibrutinib is an FDA-approved irreversible bruton’s tyrosine kinase (BTK) inhibitor used to treat CLL and MCL [19], and clinical trials of ibrutinib are undergoing for classic Hodgkin’s lymphoma (CHL) (NCT02940301) and DLBCL (NCT03399513). Although ibrutinib has shown effective inhibitory activity towards HL and NHL cell growth [56,57], ibrutinib-resistant NHL cells can develop after treatment [58]. Studies have shown that inhibiting various oncogenic pathways could be one of the strategies with which to overcome ibrutinib resistance [59,60], and multiple-agent chemotherapy is a common strategy used to treat cancers clinically, including lymphoma [61,62]. Several studies have reported that a synergistic anticancer effect was observed using a cotreatment strategy with a TrxR inhibitor (auranofin) and other clinically approved drugs in different cancer cell lines [63,64,65]. In addition, studies have shown that the phosphorylation of BTK can be regulated by ROS [66]. As mentioned previously, the ROS levels in cells are regulated by the antioxidant systems, including the Trx system. Moreover, the cytosolic Trx system and the BCR signalling pathway have a common downstream target, NF-κB, suggesting a possible correlation between these two systems.

In this study, the overexpression of mRNA and proteins of the Trx system and the BCR signalling pathway was found in the DLBCL and NHL patient samples, respectively, indicating the existence of a potential interaction between the two systems that may be involved in lymphoma survival and proliferation. For the protein analysis, normal lymph node tissue was used as a control sample. While it is common practice to use normal lymph node tissue as a control for lymphoma when assessing data from these datasets, it should be acknowledged that the lymph node contains multiple cell types, and not all of these have the potential to develop into NHL. In addition, while NHL most commonly occurs in lymph nodes it can also occur in the liver, spleen, stomach, or bones [67], and, therefore, in different tumour microenvironments. We also found that TrxR1 mRNA expression, but not Trx or PRDX1 mRNA expression, positively correlated with the mRNA expression of BTK and NF-κB, suggesting a potential interplay between TrxR, BTK, and NF-κB. The small interfering RNA experiments, although causing only approximately a 50% reduction in TrxR1 mRNA and protein levels in the SUDHL2 and SUDHL4 cells, showed that both BTK mRNA and protein levels were significantly decreased. The reduction in TrxR1 activity (~50%) obtained with lower micromolar concentrations (0.25 μM) of [Au(d2pype)_2_]Cl was associated with a significantly reduced expression of BTK, suggesting that total TrxR inhibition is not required to invoke a phenotypic effect. These results could be advantageous clinically since therapeutic interventions that only reduce TrxR expression and/or activity by 50% are less likely to kill normal healthy cells. One of the primary roles of the thioredoxin system is to regulate the cellular redox environment by maintaining healthy levels of ROS. ROS play a role as second messenger in BCR signalling [66], and the phosphorylation of BTK can be mediated by ROS in THP1 cells [68]. Increased ROS levels have been detected with increasing concentrations of [Au(d2pype)_2_]Cl treatment in both SUDHL2 and SUDHL4 cell lines [35]. While no statistically significant increase was detected at the inhibitor concentration used in the co-treatment experiments (0.25 µM), a higher concentration (1 µM) resulted in significantly increased ROS levels [35]. It is possible that changes in ROS levels due to TrxR inhibition may lead to metabolic phenotypic changes affecting BTK expression. Our findings complement those of another study, which showed that the phosphorylation of BTK can be mediated by ROS in THP1 cells [68], thus indicating that the modulation of cellular oxidants could regulate both the protein expression of BTK and its phosphorylation.

Ibrutinib is an FDA-approved BTK phosphorylation inhibitor used to treat CLL and MCL [19]. Hence, its safety and effectiveness have been proven clinically. To expand the scope of its application, ibrutinib’s anticancer activity was evaluated in the NHL cell lines. In our study, micromolar concentrations of ibrutinib alone inhibited cell proliferation in the SUDHL2 and SUDHL4 cell lines. An approximately five-fold lower IC_50_ was obtained in the SUDHL4 cells compared to that observed in the SUDHL2 cells, indicating that the GCB-DLBCL cells may be more sensitive to ibrutinib than ABC-DLBCL cells. In addition, increased sensitivity to ibrutinib was observed when the cells were treated with both [Au(d2pype)_2_]Cl and ibrutinib. The IC_50_ of ibrutinib decreased by approximately three-fold in the SUDHL2 and SUDHL4 cells. ABC (SUDHL2) and GCB (SUDHL4) are the two major subtypes of DLBCL and are characterised by different gene expression profiles, mutations, and potential clinical outcomes [69,70]. The GCB DLBCL type is similar to normal germinal centre B cells, whereas the ABC DLBCL type is similar to activated B cells [71], thus indicating their different stages of development, differentially expressed genes, and, consequently, a potential different response to treatment. It is reasonable to speculate that during treatment, different signalling pathways may be activated, including those that lead to cell proliferation and resistance to drugs. In our study, an isobologram analysis suggested the occurrence of a synergistic interaction between ibrutinib and [Au(d2pype)_2_]Cl in the two lymphoma cell lines. The combination treatment obtained a higher interaction factor value in the SUDHL2 cells, indicating that a combination treatment may be more suitable in ABC-DLBCL cells (SUDHL2) than in GCB-DLBCL cells (SUDHL4). The co-targeting of these two proteins using [Au(d2pype)_2_]Cl and ibrutinib may be more effective in this type of lymphoma, since the ABC-DLBCL subtype has high expression and constitutive activity of NF-κB [72], and both TrxR and BTK can activate the NF-κB pathway.

Our results also showed that the combination treatment of ibrutinib/[Au(d2pype)_2_]Cl could trigger apoptosis, or both apoptosis and ferroptosis, depending on the cell type. This finding showed that both lymphoma cell lines had increased caspase-3 activity after the combination treatment, and that this activity could be reversed by adding the apoptosis inhibitor. Interestingly, both apoptosis and ferroptosis inhibitors could reverse the effect of the combination treatment on the SUDHL4 cells. Ferroptosis is a well-known iron-dependent and lipid hydroperoxide-associated cell death process [73]. Antioxidant systems have been reported to play a role in regulating ferroptosis [74,75], which is a form of cell death that has been shown to be caused by an iron-dependent ROS increase [76]. However, it has also been shown that the inhibition of antioxidant systems induces apoptosis in different cancer cells [77,78]. Our previous study showed that [Au(d2pype)_2_]Cl disrupted the balance of the glutathione pool by causing GSH depletion and inhibited glutathione peroxidase activity (Gpx) activity [35]. Other studies have indicated that GSH depletion and the inhibition of GPX activity causes oxidative stress and lipid peroxidation, inducing ferroptosis [79,80], suggesting that both antioxidant systems, the thioredoxin and GSH, may have an important role to play in this type of cell death and that concurrent targeting of both systems is beneficial against cancer cells. The results in this study also showed that apoptosis and ferroptosis pathways were both activated in only the SUDHL4 cell line, suggesting that the activation of apoptosis and ferroptosis may be cell-type-specific. However, whether ferroptosis and apoptosis are interconnected is still not clear [81]. Further studies are needed to evaluate the effects of the individual compounds on ferroptosis’s mechanism of activation. In addition, since ferroptosis is iron-dependent and characterised by an accumulation of lipid ROS, both iron content and lipid peroxidation should be determined.

We further analysed the effect of the combination treatment on the BCR and NF-κB signalling pathways in the SUDHL2 and SUDHL4 cell lines. We found that both pathways were significantly affected by the combination treatment and not by the individual treatments since the mRNA expression of BTK, p65, and survivin was significantly decreased only by the synergistic effect of the two drugs. The combination treatment likely affects NF-κB signalling transduction in the SUDHL2 and SUDHL4 cells. BTK can activate IKK, thus causing the phosphorylation of IκB, and thereby activating the NF-κB pathway [82]. Therefore, using ibrutinib would lead to decreased IκB phosphorylation. A previous study using auranofin also showed the downregulation of the phosphorylation of IκB in multiple myeloma (MM) cells [83]. In this study, the downregulation of BTK protein levels was observed after the combination treatment. In addition, the decreased level of IκB phosphorylation was also found in the SUDHL2 cells, which might have resulted from the downregulation of BTK. In the research presented herein, the targeting of both BTK and TrxR caused a significant decrease in the phosphorylation of IκB through indirect inhibition with the [Au(d2pype)_2_]Cl/ibrutinib combination treatment. Moreover, the presence of NF-κB in the cytoplasm (as shown in Figure 6) indicated that the [Au(d2pype)_2_]Cl and ibrutinib cotreatment caused the inactivation of NF-κB in the SUDHL2 cells. Further investigations are needed to address the molecular mechanisms that affect the expression of the BCR pathway upon the inhibition of TrxR. The main cellular role of TrxR is to recycle the oxidised thioredoxin protein (Trx). Trx has been reported to translocate into the nucleus and regulate the binding activity of transcription factors, including NF-κB and STAT3 [84,85]. Since it has been shown that NF-κB can regulate BTK gene expression [86], it is possible that Trx may modulate the BCR signalling pathway by affecting transcription factors, such as NF-κB, that regulate BTK gene expression.

Overall, it was found that the expression of TrxR and BTK was positively correlated at both the mRNA and protein levels in DLBCL lymphoma cells, and that the inhibition of the expression of TrxR1 led to the downregulation of BTK expression. In addition, a combination treatment using a TrxR inhibitor ([Au(d2pype)_2_]Cl) and a BTK inhibitor (ibrutinib) could synergistically lead to DLBCL cell death, suggesting that co-targeting the Trx system and the BCR signalling pathway by inhibiting TrxR and BTK activity may be a novel therapeutic approach to explore further with respect to DLBCL treatment.

## Figures and Tables

**Table 1:** List of Oligonucleotides.

Protein Name	Gene Name	* Accession Number	Forward	Reverse
L32	RPL32	NC_000003.12	5′CAGGGTTCGTAGAAGATTCAAGGG3′	5′CTTGGAGGAAAACATTGTGAGCGATC3′
TrxR1	TXNRD1	NC_000012.12	5′GGAATCCACCCTGTCTCTGC3′	5′ACGAGCCAGTGGTTTGCAGT3′
BTK	BTK	NC_000023.11	5′CTGAAGAACTAAGGAAGCGGTGGATT3′	5′ACTTGTGGAGACTGGTGCTGCT3′
P65	RelA	NC_000011.10	5′ATATGAGACCTTCAAGAGCATCA3′	5′ATAGTTGATGGTGCTCAGGGATGA3′
Survivin	Survivin	NC_000011.10	5′ACCACCGCATCTCTACATTCAAGAACT3′	5′TCCCAGCCTTCCAGCTCCTT3′

* Accession number refers to the gene from which the relevant transcripts are generated.

**Table 2:** Expression levels of the Trx system and BCR signalling pathway-related proteins in NHL and healthy samples retrieved from the Human Protein Atlas.

	Normal Lymph Node	NHL
TrxR (CAB015834)	Low	High
BTK (HPA001198)	Medium	High

## Data Availability

Data will be made available upon reasonable request.

## Supplementary Materials

The following supporting information can be downloaded at: https://www.mdpi.com/article/10.3390/antiox12020529/s1. Figure S1: Expression level of the Trx system and BCR signaling pathway-related proteins in NHL and healthy samples from the human Protein Atlas. Figure S2: Effects of [Au(d2pype)2]Cl on TrxR activity in DLBCL cell lines.
