# Supplementary material for: Co-Targeting of BTK and TrxR as a Therapeutic Approach to the Treatment of Lymphoma
Source: Antioxidants (Basel). 2023 Feb 20;12(2):529. doi: 10.3390/antiox12020529 (PMC9952695; doi:10.3390/antiox12020529)
Supplement: Supplementary file 1 [file antioxidants-12-00529-s001.zip › antioxidants-2145649-supplementary.pdf]

Co-Targeting of BTK and TrxR as a Therapeutic Approach to the Treatment of Lymphoma

Sicong Wang[1,2], Erin Clapper[1,2], Kathryn F Tonissen[1,2]*, Giovanna Di Trapani[1]*

Supplementary Materials

S.1 Protein expression of TrxR and BTK in normal lymph nodes and NHL samples.

To evaluate the TrxR and BTK protein expression levels, immunohistochemistry images from Human Protein Atlas were used. The CAB015834 antibody was used to detect the TrxR expression, and the HPA001198 antibody was used to obtain the BTK expression. The results indicated low expression levels of TrxR in normal lymph nodes (Figure S.1A), and high protein expression levels of TrxR in NHL (Figure S.1B) patient samples, respectively. Although medium protein expression levels of BTK were shown in normal lymph nodes (Figure S.1C), high expression levels of BTK were observed in NHL patient samples (Figure S.1D). Overall, higher expression levels of both proteins, TrxR and BTK, were determined in NHL patient samples compared with normal lymph nodes.

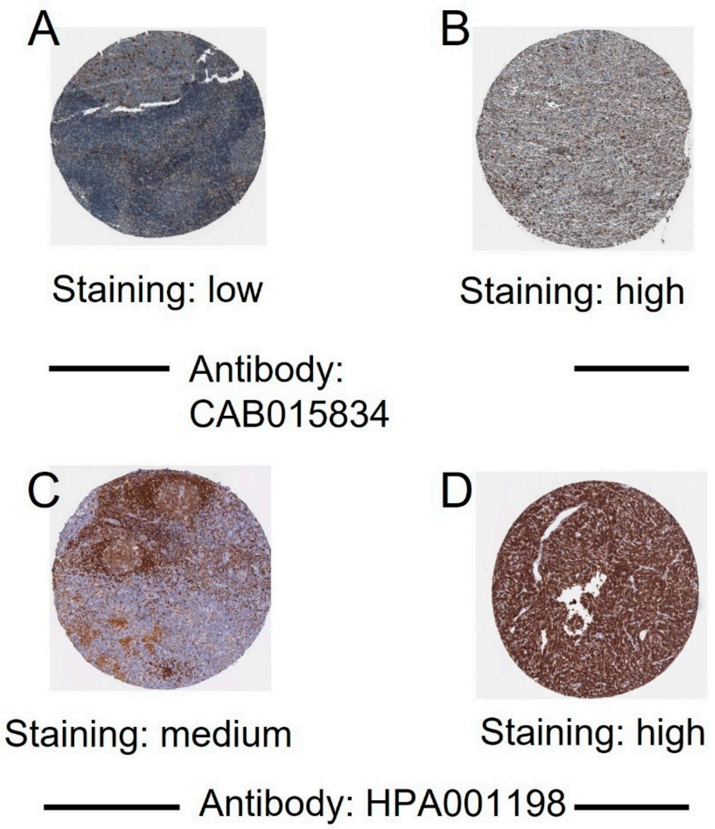

Figure S1. Expression levels of the Trx system and BCR signaling pathway-related proteins in NHL and healthy samples from the human Protein Atlas. *Representative immunohistochemistry images of TrxR1 and BTK in NHL tissues and normal lymph nodes from Human Protein Atlas (https://www.protein atlas.org). (A-B), TrxR1 expression in normal lymph node (A) and non-Hodgkin's lymphoma (B) patients' samples. (C-D), BTK expression in normal lymph node (C) and non-Hodgkin's lymphoma (D) patients' samples.*

S.2 [Au(d2pype)2]Cl inhibited the TrxR activity in the DLBCL lymphoma cell lines at a non-cytotoxic concentration.

This results section aimed to evaluate the TrxR inhibition activity of [Au(d2pype)2]Cl at a non-cytotoxic concentration to be used in the combination treatment. The results indicated that 0.25µM [Au(d2pype)2]Cl significantly decreased the activity of TrxR in SUDHL2 and SUDHL4 cells (Figure S.2A and B).

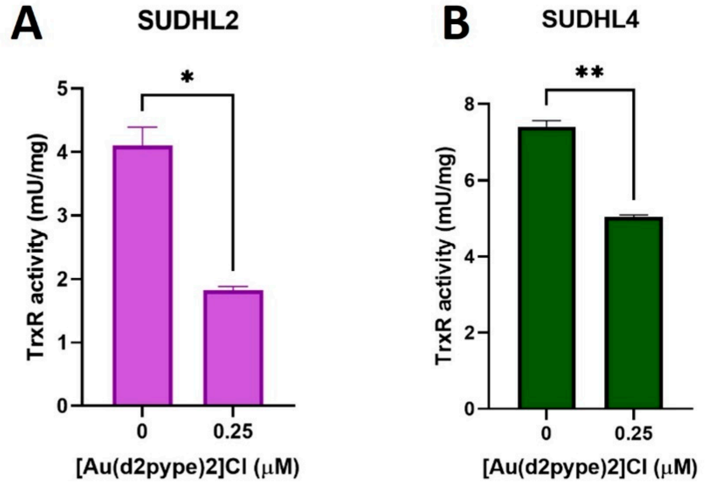

Figure S2. Effects of [Au(d2pype)2]Cl on TrxR activity in DLBCL cell lines.
*SUHDL2 and SUDHL4 cells were treated with 0.25µM [Au(d2pype)2]Cl for 24hrs. TrxR activity was measured. Results were analyzed by students' t-test. Values indicate mean ±SEM (n=3). *p<0.05, **p<0.005.*
